# Supplementary material for: Skullcapflavone II Inhibits Degradation of Type I Collagen by Suppressing MMP-1 Transcription in Human Skin Fibroblasts
Source: Int J Mol Sci. 2019 Jun 4;20(11):2734. doi: 10.3390/ijms20112734 (PMC6600147; doi:10.3390/ijms20112734)
Supplement: Supplementary file 1 [file ijms-20-02734-s001.pdf]

Supplementary Information

Skullcapflavone II Inhibits Degradation of Type I Collagen by Suppressing MMP-1 Transcription in Human Skin Fibroblasts

Young Hun Lee [1], Eun Kyoung Seo [2], and Seung-Taek Lee [1,*]

[1] Department of Biochemistry, College of Life Science and Biotechnology, Yonsei University, Seoul 03722, Republic of Korea; isop_tera@naver.com (Y.H.L.)

[2] Graduate school of Pharmaceutical Sciences, College of Pharmacy, Ewha Womans University, Seoul, 03790, Republic of Korea; yuny@ewha.ac.kr (E.K.S.)

* Correspondence: stlee@yonsei.ac.kr; Tel.: +82-2-2123-2703

Supplementary Table S1. Primer sequences used for RT-qPCR analysis of *MMP-1* and *GAPDH* mRNA expression.

**Gene**	**Nucleotide sequence**	**Nucleotide position**	**Annealing temperature (°C)**	**GenBank accession number**
MMP-1	F: 5’-GTACTGATATAATTTAGTTC-3’<br>R: 5’-GTTATCCCTTGCCTATCTAG-3’	1656-1675<br>1889-1908	45	NM_002421
GAPDH	F: 5’-ACTGCTTAGCACCCCTGGCCA-3’<br>R: 5’-TTGGCAGTGGGGACACGGAAG-3’	488-508<br>720-740	62	BC023632

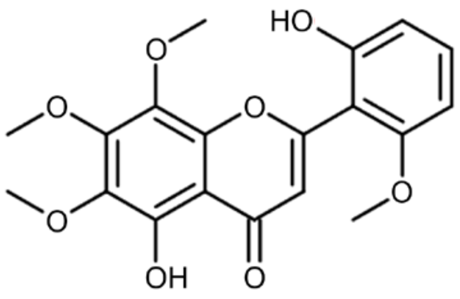

Supplementary Figure S1. Chemical structure of skullcapflavone II (5-hydroxy-2-[2-hydroxy-6-methoxyphenyl]-6,7,8-trimethoxychromen-4-one).

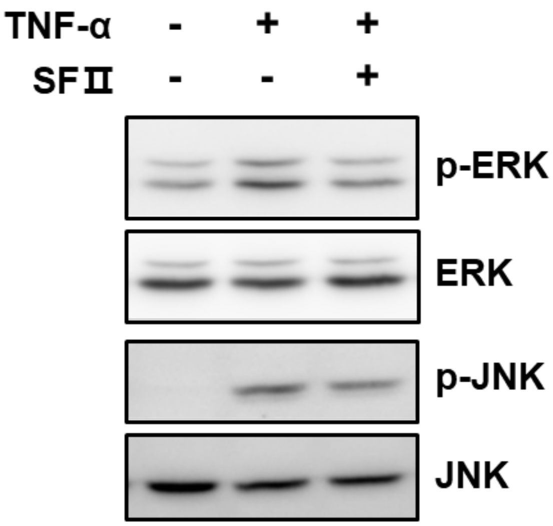

Supplementary Figure S2. Effect of skullcapflavone II on TNF-α–induced phosphorylation of ERK and JNK in foreskin fibroblasts.

Subconfluent foreskin fibroblasts were starved for 24 h in a serum-free medium, pre-incubated with (+) or without (-) 3 μM skullcapflavone II for 30 min in a serum-free medium, and stimulated with (+) or without (-) 1 ng/ml TNF-α for 10 min. Cell lysates were prepared and analyzed by Western blot using antibodies against the indicated molecules.
